# Occupational Contact Dermatitis in Italy: Bridging the Gap Between Estimated Incidence and Official Reporting Data

**DOI:** 10.3390/jcm15187330

**Published:** 2026-09-21

**Authors:** Sara Scilli, Alberto Modenese

**Affiliations:** Department of Biomedical, Metabolic and Neural Sciences, University of Modena & Reggio Emilia, 41125 Modena, Italy; 253844@studenti.unimore.it

**Keywords:** contact dermatitis, allergic, irritant, occupation, prevention

## Abstract

**Background/Objectives**: Occupational Contact Dermatitis (OCD) is one of the most common occupational diseases and represents a substantial clinical and socioeconomic burden. Despite its high prevalence, OCD is frequently underrecognised and underreported, limiting the effectiveness of occupational health surveillance and preventive strategies. This study aimed to investigate the extent of OCD underreporting in Italy by comparing official reporting data with incidence estimates derived from epidemiological data available in the scientific literature. **Methods**: Official data on dermatitis and eczema cases reported to the Italian National Institute for Insurance against Accidents at Work (INAIL) between 2020 and 2024 were analysed and compared with incidence estimates obtained through a systematic literature search of epidemiological studies, surveillance systems, and cohort studies conducted in high-risk occupational groups with available specific incidence data in the scientific literature, such as healthcare workers, construction workers, and hairdressers. **Results**: INAIL data showed an average of approximately 182 reported OCD cases per year during the study period. However, the estimates we calculated suggested a substantially higher disease burden, with an annual estimate ranging from 1439 cases to 21,412 cases. This implies that, depending on the incidence source used, only 0.8% to 12.7% of the expected cases are notified. **Conclusions**: OCD appears to be substantially underreported in Italy. Underrecognition of its occupational origin, the low severity often attributed to symptoms, and the limits of current surveillance systems likely contribute. Better occupational health surveillance, greater awareness among clinicians and workers, and simpler reporting procedures would improve case recognition and help prevent progression to chronic disease.

## 1. Introduction

Occupational skin diseases represent one of the most common categories of work-related illnesses worldwide, accounting for up to 30–45% of all occupational diseases in several settings [1]. Among these, Occupational Contact Dermatitis (OCD) is by far the most prevalent condition, contributing to the vast majority of cases and representing a significant clinical and socioeconomic burden [2].

Contact dermatitis is an inflammatory skin disorder caused by exposure to exogenous substances, typically classified into Irritant Contact Dermatitis (ICD), resulting from direct cytotoxic effects on the skin barrier, and allergic contact dermatitis (ACD), which is mediated by a delayed type IV hypersensitivity reaction [3]. Occupational exposures to chemical, physical, and biological agents make workers particularly vulnerable, especially in high-risk sectors such as healthcare, construction, manufacturing industries, and hairdressing [4].

Although OCD is widely recognised as one of the leading occupational diseases, its actual burden remains difficult to estimate accurately. Surveillance systems based on compensation claims consistently underestimate the real incidence, capturing only a fraction of clinically diagnosed cases [2]. Studies conducted across Europe and beyond have identified multiple barriers to OCD notification, including organisational constraints, limited awareness among workers and physicians, perceived low severity of symptoms, and structural limitations of compensation systems [5,6]. These challenges appear to be universal and are not restricted to any single health system [7].

In Italy, occupational disease surveillance relies primarily on data from the Italian Workers’ Compensation Authority (Istituto Nazionale per l’Assicurazione contro gli Infortuni sul Lavoro—INAIL). The available figures suggest a considerably restricted case pool compared to other European countries: INAIL statistical data indicate that skin and subcutaneous tissue diseases account for approximately 300 recognised cases per year, representing 1.3% of all positively assessed cases of occupational diseases (i.e., all localisations and exposure agents) in the 2015–2019 quinquennium. Furthermore, a declining trend has been observed, with skin diseases falling from 5% of work-related conditions in the early 2000s to approximately 1% in recent years. A key missed opportunity lies in the fact that skin disease diagnoses in Italy are rarely linked to occupational history at the time of clinical assessment, which prevents the identification of work-related exposures that may have caused or exacerbated the condition [8].

Despite this context, the magnitude of OCD underreporting in Italy has not been systematically quantified. This study aims to address this gap by comparing five years of INAIL records (2020–2024) with the expected incidence rates derived from the literature.

## 2. Materials and Methods

This article reports an ecological study based on secondary data, in which the expected number of OCD cases in Italy was modelled by applying published occupational incidence rates to national workforce denominators and then compared with the cases officially reported to INAIL over the same period.

### 2.1. Identification of Relevant Scientific Literature Data on the Incidence of OCD

Incidence estimates were obtained through a structured, systematic literature search aimed at identifying the most appropriate published incidence data.

PubMed/MEDLINE was searched using the following string: (“occupation*” AND “dermatitis” AND “incidence”) AND (“UK” OR “Europe*” OR “Ital*”). The search returned 147 records; the 114 records published within the preceding 30 years (1997–2026) were screened by title and abstract against the eligibility criteria below. Records not providing an incidence rate applicable to a workforce denominator and records outside the European/UK setting were excluded at this stage.

Inclusion criteria:

1. Reference sources (including reviews) reporting OCD incidence rates disaggregated across multiple occupational or industrial categories in the Italian or other European (including the UK) workforce.
2. Original studies (reviews excluded) conducted in Italy or another European country (including the UK) that estimated OCD incidence, longitudinally or retrospectively, within a defined occupational cohort; one study was retained per cohort.

Where the same cohort had been investigated in more than one eligible study, an Italian study was preferred. Where no Italian study was available, the most recent study (published within the preceding 15 years) conducted in the geographically closest European setting was retained. Where no study meeting these preferences was available for a given cohort, the most pertinent older study within the 30-year search window was used.

Exclusion criteria:

1. Studies estimating incidence specifically in the context of COVID-19-related co-exposures (e.g., pandemic-related changes in hygiene practices or personal protective equipment use) to avoid distortion of baseline occupational incidence rates.
2. Studies estimating only the incidence of contact dermatitis specifically linked to a single chemical agent involved in the occupational exposure of the work sector considered.

We selected two main sources of data according to inclusion criterion 1: the UK EPIDERM study and the German study by Diepgen et al. [9,10].

EPIDERM is one of the four voluntary reporting schemes that collect data in the UK surveillance scheme for work-related skin diseases (i.e., THOR-UK) [11]. EPIDERM provides sector-specific incidence rates based on reports from both consultant dermatologists and occupational physicians, organised according to a standard classification of economic activities used in the UK [12] and derived from ISIC [13], as is also the case for the classification adopted in Italy (i.e., ATECO) [14] and used by INAIL for workers’ compensation purposes. A notable feature of the EPIDERM system is that incidence estimates differ depending on the two reporting sources (i.e., dermatologists vs occupational physicians). Both sets of incidence rates were therefore extracted from EPIDERM and are reported in Table 1 [9].

Similarly, incidence rates for 13 occupational categories were extracted from the study by Diepgen et al. and are reported in Table 2 [10]. This source was retrieved through the reference list of a review identified by the literature search, which reports it as the epidemiological reference for European occupation-specific incidence data [15].

In addition to data from EPIDERM and Diepgen, suitable for many different occupational activities in Europe, within the same literature search we identified further studies reporting incidence in specific occupational cohorts, selected according to inclusion criterion 2. For three cohorts (construction workers, healthcare personnel, and hairdressers) recent studies conducted in Italy were available [16,17,18].

For construction workers, an incidence rate of 2.7 cases per 10,000 workers in 2016 (the most recent year available) was derived from a retrospective patch-test database study of construction workers in North-Eastern Italy [18]. For healthcare workers, an incidence rate of 45 per 10,000 workers per year was derived from a 10-year prospective cohort study conducted in North-Eastern Italy [16]. For hairdressers, a retrospective study in North-Eastern Italy reported a mean incidence from 1999 to 2016 of 7.4 cases per 10,000 workers per year [17].

For two further sectors, no recent Italian or European data were available, and the most pertinent older sources were used. For food manufacturing, a seven-year UK company health-surveillance programme reported a mean incidence of 13.1 cases per 10,000 employees/year, of which 96% were irritant contact dermatitis [19]. This overall rate was applied in Table 3, while the same source provided separate rates for bread bakers (17.0 per 10,000/year) and cake bakers (7.7 per 10,000/year) used in Table 4. For mechanics, an older Italian regional register provided an incidence of 1.9 cases per 10,000 workers/year, used solely for this category (more recent Italian studies from the same research group covered the other occupational categories considered) [20].

### 2.2. Quality Assessment of the Incidence Sources

The methodological quality of the selected incidence sources was appraised using an adaptation of the methodology proposed in Jensen 2008 [21] and already applied by Della Vecchia et al. [22]. The quality of the studies, their strengths and weaknesses, were evaluated by the authors according to five dimensions: study design and materials, consideration of possible confounders, methods for outcome measurements, methods for exposure assessment, and modalities of data presentation and statistical analysis. Each dimension was assigned a score from 0 to 3, for a maximum of 15, and sources were graded as low (+), medium (++), or high (+++) quality (Table A2). Two authors scored the sources independently, with disagreements resolved through discussion.

### 2.3. Estimates of the Number of Exposed Workers in Italy and Calculations of the Expected OCD Incidence in Italy

We consulted the publicly accessible INAIL statistical database (BDS—Banca Dati Statistica) for the period 2020–2024 [23].

For the EPIDERM incidence estimates based on the number of UK workers employed in the economic activities classified according to the UK-SIC, we first derived the corresponding number of Italian workers employed in the same economic sector in the year 2024 and then applied the incidence rates to these numbers in order to retrieve the expected OCD cases in Italy [9]. We calculated further specific estimates for the healthcare and construction sectors based on the more recent European incidence data available through the two longitudinal studies identified [16,18].

Moving from OCD incidence rates per workers in economic activities to rates per specific occupation, we considered the data by Diepgen et al. [10], and to obtain a proper, if approximate, comparison, we disaggregated the subcodes of economic sectors in the INAIL database in order to retrieve the number of Italian workers actually occupied in the specific job task in 2024. The correspondence between Diepgen’s occupational categories and the Italian classification of economic activities sub-codes is reported in Appendix A, Table A1. The expected OCD cases in Italian workers were then calculated by applying the German incidence rates. For healthcare workers and hairdressers, we also considered the available Italian estimates [16,17].

### 2.4. Evaluation of the Underreporting of OCD in Italy

In the BDS, which by definition records only conditions notified as occupational in origin, claims are grouped by ICD-10 codes. Among the ‘Diseases of the Skin and Subcutaneous Tissue’ (L00–L99) codes, we selected the subcategory ‘Dermatitis and Eczema’ (L20–L30), which includes contact, atopic, and seborrhoeic dermatitis. As the modelled outcome is occupational contact dermatitis, the numerator was restricted to contact-dermatitis entries only. During 2020–2024, the contact-dermatitis subset averaged approximately 182 cases/year (2020: 164; 2021: 173; 2022: 191; 2023: 178; 2024: 203); we compared this number to the estimated expected cases in order to assess the extent of underreporting.

## 3. Results

### 3.1. Estimated Incidence of OCD in Italian Workers by Economic Activity

The estimated annual cases of OCD in Italian workers grouped by economic activity are shown in Table 3. The overall number of expected OCD cases per year ranged from approximately 1439 cases when applying EPIDERM rates from dermatologists to an upper estimate of 21,412 cases when applying EPIDERM incidence rates derived from occupational physicians integrated by the two recent longitudinal studies on healthcare and construction workers we identified. The lowest number of cases is expected in the agriculture, forestry, and fishing sector, employing more than 52,000 workers in Italy and resulting in expected crude cases ranging from 6 to 19 diseases reported per year. On the other hand, the highest number is observed in the metallic and automotive product manufacturing sector, employing almost 2.8 million workers in Italy, with the expected cases of OCD ranging from 389 to 10,343 (Table 3).

### 3.2. Estimated Incidence of OCD in Italy by Specific Job Tasks

The estimated annual OCD cases in Italy for each occupational category identified by Diepgen et al. were calculated by applying the corresponding incidence rates to the number of Italian workers employed in each occupation in 2024 (Table 4) [10]. For four categories, incidence estimates from additional studies were included alongside the German rates: healthcare workers, hairdressers, mechanics, and bakers, reporting separate rates for bread and cake baking [16,17,19,20]. Rates for bread and cake bakers derive from the corresponding sub-sectors in Smith 2004 [19]; as the Italian workforce is not disaggregated into two groups, each rate is applied to the whole baker category as an alternative scenario. Considering the 13 occupational categories identified by Diepgen et al. [10], integrated with the two available Italian studies on healthcare workers and hairdressers, the total estimated number of expected OCD cases in Italy resulted in a range varying between 3663 and 13,578 cases per year, affecting a workforce of approximately 3.2 million workers (Table 4).

### 3.3. Estimate of the Underreporting of OCD in Italy

INAIL reported an average of 182 OCD cases/year between 2020 and 2024 across all sectors. The minimum number of expected OCD cases in Italy to be reported to the compensation authority was obtained by applying EPIDERM incidence rates based on dermatologists’ notifications by economic activity. Accordingly, in the best-case scenario, only 12.7% of incident cases are actually reported to INAIL. Our upper estimate resulted in over 21,000 expected OCD cases per year in Italian workers, therefore representing the worst-case scenario with only 0.8% of the diseases notified.

## 4. Discussion

The present study provides a scenario-based quantification of OCD underreporting in Italy, revealing a marked discrepancy between officially recorded and epidemiologically estimated cases. This gap is relevant as it may reflect a systematic failure to recognise and document a disease that, when left unaddressed, tends to progress and become increasingly difficult to manage [5,24].

This gap has several plausible explanations, most of them related to how mild cases are managed. OCD is frequently perceived by affected workers and sometimes even by their physicians as a minor or self-limiting condition rather than a compensable occupational disease. In early-stage cases, workers commonly self-manage with emollients or over-the-counter preparations, consult a general practitioner who may not consider the occupational dimension or simply change jobs without any formal notification ever being filed [25,26].

The diagnosis of OCD can be relatively straightforward, being based on anamnesis, clinical presentation and specific tests, such as patch testing for ACD. Nevertheless, some forms may go unnoticed when they affect workers apparently not exposed to occupational risk factors, when clinical manifestations and symptoms are mild, or when they occur in subjects with predisposing conditions, such as atopic dermatitis, or with concomitant non-occupational dermatoses. A proportion of cases is inevitably misdiagnosed, since clinical pattern and severity alone do not reliably indicate the presence or the relevance of a contact allergy [25,27]. Underreporting is also possible when acute forms of OCD, especially ICD, are managed as injuries rather than diseases [28].

Classical OCD typically develops over months or years of cumulative exposure, and the clinical manifestation usually gives only little indication of the actual occupational risk factor involved. Moreover, it has to be considered that various occupational factors, especially chemicals, may induce a synergistic effect. Physical and environmental factors are also relevant: wet work is the main cause of ICD and a relevant co-factor for ACD, and the risk increases with the duration and frequency of exposure, as prolonged or repeated contact with water combined with irritant and allergenic chemicals favours the development of dermatitis in occupations such as hairdressing and cleaning [27,29,30].

Work-related psychosocial factors may also contribute to the occurrence and severity of these chronic occupational dermatoses and their impact on occupational activity [31].

Reliable, complete OCD notification data allow preventive resources to be directed where they are most needed, ultimately reducing health risks for workers and the clinical impact of the disease. Where transparent, easily accessible notification procedures have been coupled with incentives for early reporting, the clinical impact of the disease has been reduced, with a documented decrease in disease-related costs and job losses [25]. Workplace prevention mainly relies on complete removal, when possible, or at least on the reduction to the lowest level possible of offending allergens or irritants to avoid or reduce potentially harmful contact. Wet work also needs to be minimised whenever possible. In high-risk sectors, such as hairdressing and healthcare, structured skin-protection and education programmes, together with preventive and periodic occupational health surveillance and dermatological screening, should be implemented [29]. At the individual level, in addition to personal protective equipment as primary prevention, secondary and tertiary prevention also need to be considered. Early diagnosis and treatment of OCD and interruption of exposure for workers who develop the disease are fundamental measures. Moreover, useful interventions are those dedicated to the restoration of the epidermal barrier with emollients, and those dedicated to the control of inflammation with topical corticosteroids [24]. Finally, timely notification, treatment, and compensation of the work-related disease also favour an appropriate return to work once the condition has been adequately managed [25,27].

Unfortunately, our study suggests that OCD in Italy is largely underreported: even in the most conservative scenario, the vast majority of expected cases never reach the workers’ compensation authority. Nevertheless, due to the limitations of our study, specifically discussed in the following sub-paragraph, the range of our underreporting estimates is quite wide: the two bounds we obtained, i.e., a number of notified cases of t% up to 12.7% of the total expected OCD cases, are only a proxy for the real reporting rate, which is likely to fall between them. As a general note, it should be considered that the underreporting of occupational diseases is not limited to OCD, and it is unfortunately widely spread and can affect different diseases, including occupational cancers, work-related psychological and psychiatric disorders and many others [32]. It appears that both workers and physicians are somehow disincentivised to report the diseases. Among the main reasons for workers, there is the fear of losing the job and the concerns related to workplace relations; for the physicians, the main reasons are related to the administrative complexity of the notification process and to the lack of time, as these activities require additional time after the usual clinical activity [6,33].

### 4.1. Study Limitations

The estimates of the incidence of OCD in Italian workers provided in this analysis have several limitations, as listed below:

1. Some of the incidence estimates applied are relatively dated and relate to occupational settings whose risk factors may no longer have the same impact today (e.g., latex exposure in the Healthcare sector);
2. The differences in OCD case definitions, diagnostic criteria, ascertainment methods, and surveillance systems may have affected the comparability and the estimated magnitude of underreporting;
3. OCD incidence estimates to be applied to the number of exposed workers in Italy have been retrieved through a systematic literature search, even if it involved only the PubMed-MedLine database, possibly introducing a selection bias;
4. Most of the data used to derive the Italian incidence estimates of OCD come from other European countries (particularly the UK and Germany), where the main work-related risk factors involved may have different distributions and relevance compared to Italy;
5. EPIDERM data are based on economic activities rather than on specific occupations. Similarly, for the Italian data we considered the total number of workers employed in each economic activity, although not all of them are necessarily exposed to the risk factor involved in the onset of the skin disease. This may lead to an overestimation or underestimation of the real incidence, depending on the different distribution of exposed vs non-exposed workers within the same economic sectors in the two Countries (the UK vs Italy);
6. Apart from construction workers, health personnel, bakers, mechanicians, and hairdressers for whom quite recent European data have been retrieved, other specific occupation-based incidence data of OCD useful for our Italian estimates were available only for a limited number of professions through the German study of Diepgen et al. [10] published more than 25 years ago;
7. When applying occupation-based incidence rates of OCD to Italian workers, we realised that the numbers of subjects specifically engaged in the particular job task were not officially and publicly available for Italy. To retrieve these numbers, we further disaggregated the economic activities classification data, looking for the number of workers employed in sub-codes of activities (Appendix A, Table A1), possibly resulting in an overestimation of the exposed workforce for a given job task and, consequently, of the expected cases. Nevertheless, we have also to say that this limitation did not actually affect our lower and upper OCD incidence estimates for Italy and, accordingly, our evaluation of the extent of underreporting. In fact, both of the extremities of the incidence rate range have been obtained after the application of the EPIDERM study rates, which are based on economic activity classification rather than job tasks. Our lower estimates are based on the EPIDERM notifications from dermatologists, while the upper ones derive from the reporting of the occupational physicians (integrated with the incidence data shown in the additional sector-specific incidence studies on food manufacturing, construction, and healthcare sectors);
8. It should be taken into account that possibly a few cases reported in the EPIDERM or Diepgen et al. [10] studies as occupational diseases may have fulfilled in Italy the criteria for notification as occupational injuries. Unfortunately, more precise estimates are not possible as publicly available INAIL data on occupational injuries do not report any ICD classification;
9. Because confidence intervals and raw data were not consistently available across the selected incidence sources, a formal probabilistic uncertainty analysis could not be performed; therefore, uncertainty was addressed through deterministic scenario-based estimates using alternative incidence sources;
10. As a final limitation, it has to be highlighted that the quinquennium 2020–2024 for which INAIL data on the reported occupational diseases are available largely overlaps with the COVID-19 pandemic, which affected both the true incidence of OCD and notification activity; the resulting temporal pattern was examined in the following paragraph.

### 4.2. Impact of the COVID-19 Pandemic

The number of OCD claims was lowest in 2020 (164) and was highest in 2024 (203), a pattern that is unlikely to reflect real incidence. Indeed, the pandemic period involved intensified hand hygiene and prolonged use of personal protective equipment, both known to increase irritant contact dermatitis [34]. A plausible explanation is that reduced access to occupational health services during the lockdowns depressed notifications, while true disease frequency was rising, with the subsequent recovery through 2024 reflecting renewed reporting activity.

## 5. Conclusions

Our exploratory estimates indicate that official reports likely capture only a small fraction of the OCD affecting Italian workers. The most immediate response lies in clinical routine: taking an occupational history in any patient presenting with hand or forearm dermatitis is a frequently missed step that helps establish the occupational origin of the condition, and patch testing, applying profession-specific series, should also follow whenever an allergic component is suspected. At the same time, occupational health surveillance should include dermatological consultation to screen workers in high-risk sectors, improving early disease detection.

In the longer term, Italy would benefit from a population-level surveillance system for OCD, providing more reliable incidence data than those currently available and laying the foundations to plan workplace preventive interventions and to evaluate their impact over time.

## Figures and Tables

**Table 1:** EPIDERM incidence rates of OCD (cases per 10,000 workers/year) by classified group of economic activities, based on reports from dermatologists and occupational physicians.

Industrial Categories Considered in the EPIDERM Study	EPIDERM Incidence, Based on Reporting of:	
	Dermatologists	Occupational Physicians
Agriculture, forestry, and fishing	1.20	3.69
Mining and quarrying	1.19	13.70
Food and organic material manufacturing	1.01	5.74
Petrochemical, rubber, and plastic manufacturing	1.53	18.40
Metallic and automotive product manufacturing	1.39	36.94
Utilities and Construction	0.71	2.54
Health and social services	1.09	3.42
Others	0.44	1.48

**Table 2:** OCD incidence rates (per 10,000 workers/year) by occupational category.

Occupational Categories Considered by Diepgen et al. [10]	OCD Incidence Rate (per 10,000 Workers/Year)
Hairdresser	194
Baker	64
Electroplaters	38
Florist	34
Confectioner	28
Tile setters	25
Plumber	19
Metal, Surface worker	13
Dental technician	12
Cooks	11
Health service	11
Mechanician	8
Leather worker	8

**Table 3:** Expected annual OCD cases in Italy by economic activity, based on EPIDERM incidence rates and European cohort studies in the healthcare and construction sectors.

Economic Activity	Workers Employed in Italy (2024)	Expected OCD Cases According to the Incidence From:		
		EPIDERM, Based on Reporting of:		Radillo et al.; Larese et al.; Smith et al. [16,18,19]
		Dermatologists	Occupational Physicians	
Agriculture, forestry, and fishing	52,822	6.34	19.49	
Mining and quarrying	21,442	2.55	29.38	
Food and organic material manufacturing	1,126,356	113.76	646.53	1475.53
Petrochemical, rubber, and plastic manufacturing	566,278	86.64	1041.95	
Metallic and automotive product manufacturing	2,799,869	389.18	10,342.72	
Utilities	456,788	32.43	116.02	
Construction	1,810,437	128.54	459.85	488.82
Health and social services	1,833,877 (1,396,835 in Health service)	199.89	627.19	6285.76 *
Others	10,892,959	479.29	1612.16	
Total	19,560,828	1438.63	14,895.28	
Total number of expected cases		Lower estimate: 1438.63	Upper estimate: 21,411.82	

* Expected OCD cases based on health services only.

**Table 4:** Expected annual OCD cases in Italy by specific occupational category, based on incidence rates from Diepgen et al. [10] and additional sector-specific incidence studies of European occupational cohorts.

Occupational Category	Workers Employed in Italy (2024)	Expected OCD Cases According to the Incidence From:	
		Diepgen et al. [10]	Larese et al.; Piapan et al.; Smith et al. [16,17,19,20]
Hairdresser	222,597	4318.38	164.72
Bread Baker	158,001	1011.21	268.60
Cake baker			121.66
Electroplaters	50,858	193.26	
Florist	10,184	34.63	
Confectioner	45,031	126.09	
Tile setters	35,212	88.03	
Plumber	204,947	389.40	
Metal-, Surfaceworker	153,840	199.99	
Dental technician	14,475	17.37	
Cooks	628,440	691.28	
Health service	1,396,835	1536.52	6285.76
Mechanician	201,596	161.28	38
Leather worker	76,638	61.31	
Total	3,198,654	8828.75	/
Expected OCD cases in Italy:	Min. 3662.56–Max. 13,577.98		

## Data Availability

INAIL data are publicly available at https://bancadatistatisticaoas.inail.it/analytics/saw.dll?Dashboard, accessed on 17 July 2026. All epidemiological data used for incidence estimates are available in the cited published references.

## Abbreviations

The following abbreviations are used in this manuscript:

OCD	Occupational Contact Dermatitis
ICD	Irritant Contact Dermatitis
ACD	Allergic Contact Dermatitis
INAIL	Istituto Nazionale per l’Assicurazione contro gli Infortuni sul Lavoro (Italian Workers’ Compensation Authority)
EPIDERM	UK Occupational Skin Disease Surveillance scheme
ICD-10	International Classification of Diseases, Tenth Revision
ATECO	Attività Economiche (i.e., Italian classification of economic activities)
ISIC	International Standard Industrial Classification
UK-SIC	Standard Industrial Classification, United Kingdom
BDS	“Banca Dati Statistica”, the INAIL statistical database

## Appendix A

### Appendix A.1

**Table A1:** Correspondence between occupational categories at risk for OCD (Diepgen et al. [10]) and the Italian ATECO classification codes, with the description of the activity reported in the INAIL database.

Occupational Categories (Diepgen et al. [10])	Italian ATECO Sub-Code	Description of the Occupational Activity According to the Italian ATECO Sub-Code
Hairdresser	S9602	Hairdressing and other beauty treatment services
Baker	C107	Manufacture of bakery and farinaceous products
Electroplaters	C2561	Metal treatment and coating
Florist	G47761	Retail sale of flowers and plants
Confectioner	I56103	Ice cream parlours and pastry shops
Tile setters	F43330	Floor and wall covering
Plumber	F43220	Installation of plumbing, heating, and air conditioning systems (including maintenance and repair)
Metal, Surface worker	C2562	General mechanical engineering works
Dental technician	C32502	Manufacture of dental prostheses (including repair)
Cooks	I561–I562	Restaurants and mobile food service activities-catering and other food service activities
Health service	Q86	Healthcare
Mechanician	G4520–G45403	Maintenance and repair of motor vehicles; maintenance and repair of motorcycles and mopeds (including tyres)
Leather worker	C151	Preparation and tanning of leather; manufacture of travel goods, handbags, saddlery, and harness; dressing and dyeing of fur

### Appendix A.2

**Table A2:** Quality assessment of the studies included in the review. Quality score: 0–5 = + (low); 6–10 = ++ (medium); 11–15 = +++ (high).

Authors, Year	Design and Materials	Consideration of Potential Confounders	Measurement of Outcome	Measurement of Exposure	Data Presentation and Statistical Analysis	Quality Assessment Score	Total Score
McDonald et al., 2006 [9]	2	2	3	1	2	10	++
Diepgen et al., 1999 [10]	1	2	2	2	3	10	++
Larese Filon et al., 2017 [16]	3	3	3	3	3	15	+++
Radillo et al., 2021 [18]	2	2	3	2	3	12	+++
Piapan et al., 2020 [17]	2	2	3	2	3	12	+++
Larese et al., 2003 [20]	2	2	2	2	2	10	++
Smith et al., 2004 [19]	2	2	2	2	2	10	++
